# Robot‐Aided Measurement of Insect Diversity on Vegetation Using Environmental DNA

**DOI:** 10.1002/ece3.71391

**Published:** 2025-05-07

**Authors:** Darina Koubínová, Steffen Kirchgeorg, Christian Geckeler, Sarah Thurnheer, Martina Lüthi, Théophile Sanchez, Stefano Mintchev, Loïc Pellissier, Camille Albouy

**Affiliations:** ^1^ Ecosystems and Landscape Evolution, Department of Environmental Systems Science ETH Zürch Zürich Switzerland; ^2^ Swiss Federal Institute for Forest Snow and Landscape Research WSL Birmensdorf Switzerland; ^3^ Environmental Robotics Laboratory, Department of Environmental Systems Science ETH Zürich Zürich Switzerland

**Keywords:** drones, environmental DNA, insects, metabarcoding, non‐invasive biodiversity monitoring, Oxford nanopore sequencing, vegetation swabbing

## Abstract

Traditional methods of biodiversity monitoring are often logistically challenging, time‐consuming, require experienced experts on species identification, and sometimes include destruction of the targeted specimens. Here, we investigated a non‐invasive approach of combining the use of drones and environmental DNA (eDNA) to monitor insect biodiversity on vegetation. We aimed to assess the efficiency of this novel method in capturing insect diversity and comparing insect composition across different vegetation types (grassland, shrub and forest) in Switzerland. A commercial, off‐the‐shelf drone was equipped with a specialised probe that autonomously swabbed vegetation and collected eDNA. Then, samples were processed using rapid third‐generation Oxford Nanopore sequencing. The obtained data were analysed for insect diversity, comparing taxonomic richness, evenness and community composition across the three habitat types using statistical techniques. Sequencing of the samples yielded 76 hexapod taxa, revealing an insect community with notable differences in taxonomic richness but not in evenness across grassland, shrub and forest habitats. Our study demonstrates the potential of drone‐based sampling integrated with eDNA and nanopore sequencing for biodiversity monitoring, offering a non‐destructive method for detecting insect occurrence on plant surfaces. Integrating robotics and eDNA technology provides a promising solution for fast, large‐scale, non‐invasive biodiversity monitoring, potentially improving conservation efforts and ecosystem management.

## Graphical abstract

We developed a novel method combining drone‐based sampling and environmental DNA (eDNA) with Oxford Nanopore sequencing to monitor insect biodiversity on vegetation. Using a DJI Matrice 3 drone, we collected eDNA samples from grassland, shrub and forest habitats in Switzerland, detecting 64 insect taxa and revealing habitat‐specific differences in diversity. This approach offers a non‐invasive, scalable solution for biodiversity monitoring, enhancing conservation and ecosystem management efforts.

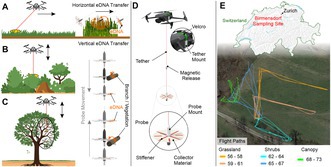

## 1 Introduction

Grasslands and shrublands, shaped by arid conditions or management practices that limit tree dominance, often support remarkable biodiversity by fostering a mosaic of habitats ideal for numerous plant and animal species (Dengler et al. 2014; DiPaolo 2020; Petermann and Buzhdygan 2021). Within these landscapes, open semi‐natural grasslands combined with shrub patches offer an environment that supports a high diversity of plant species that form the foundation of complex food webs, sustaining a diverse community of insects and other organisms (Blair et al. 2013; Debinski 2023). However, overexploitation through practices such as overgrazing, irrigation and fertilisation has led to a significant decline in habitat quality, resulting in a loss of species diversity within these ecosystems, particularly observed in Europe (Boch et al. 2021; Dengler et al. 2020). Similarly, forests, characterised by their complex vertical and heterogeneous structure, support a high level of plant and animal diversity (Filotas et al. 2014), but are under pressure from logging, conversion, degradation, fragmentation, pest species or climate change (Leuschner and Homeier 2023). In response, efforts to conserve and restore grasslands, their adjacent shrublands, and forests have increased in many regions (Lyons et al. 2023; Van Vooren et al. 2018; Wuepper et al. 2024). Despite these initiatives, there remains a critical need for efficient tools to evaluate the effectiveness of conservation efforts over time and whether the biodiversity in these ecosystems is in a good state and evolves positively over time (Buřivalová et al. 2023; Török et al. 2021).

While monitoring is often conducted at the onset of management projects to conserve or increase biodiversity, the high costs associated with long‐term monitoring can hinder continued assessments, leaving gaps in our understanding of ecosystem recovery and improvement (Caughlan and Oakley 2001). Since subsidies can be provided to farmers, forest owners and managers in certain regions as part of these conservation efforts, for example, for the maintenance of grasslands, shrubland and forest quality for biodiversity (Bratu et al. 2024; Mann et al. 2023), adequate efficient monitoring is needed to measure the long‐term success of these initiatives (Reboud et al. 2021; Stephenson et al. 2022). The monitoring and measurement of biodiversity are thus crucial for assessing the variety of organisms present in specific ecosystems and more effectively targeting conservation efforts (Moersberger et al. 2024). Traditional methods of monitoring involve vegetation surveys and direct observation, call identification, trapping, sign surveys or manual collection of animals (Sutherland 2006). However, these methods are often logistically challenging, time‐consuming and require extensive expertise for accurate species identifications, especially in taxonomically complex groups. Therefore, monitoring (managed) ecosystems requires the development of modern methods that can be easy to implement, economical and time‐effective.

Environmental DNA (eDNA) metabarcoding is a powerful tool for assessing biodiversity within a specific area with the possibility to detect even rare species which may remain undetected by using conventional methods (McElroy et al. 2020; Ruppert et al. 2019). eDNA from water is particularly valuable because it can integrate signals from the entire catchment area, providing a broad assessment of biodiversity across a large region, making it a cost‐effective approach (Prié et al. 2023; Reji Chacko et al. 2023). In the absence of water bodies, finding an alternative sampling method is challenging, and attempts have been made with soil or air sampling (Johnson et al. 2021; Kirse et al. 2021; Lynggaard et al. 2022; Oliverio et al. 2018). Soil samples tend to provide highly localised information, while air sampling generally provides weaker signals and requires prolonged filtration to capture sufficient DNA (Edwards et al. 2018; Polling et al. 2024; Roger et al. 2022). Recent studies suggest that a significant amount of eDNA accumulates on plant leaves or flowers, potentially offering a more robust signal than air samples (Gamonal Gomez et al. 2023; Kudoh et al. 2020; Lynggaard et al. 2023; Yoneya et al. 2023). Over time, leaves, flowers or other parts of the plants can collect and retain DNA from various sources, making them an excellent medium for eDNA sampling (Johnson et al. 2023; Krehenwinkel et al. 2022). By swabbing the leaves, it is possible to manually collect substantial quantities of DNA (Lynggaard et al. 2023). Swabbing manually on large surfaces can be labour‐intensive and impractical for covering large areas, presenting a challenge for wide‐scale biodiversity monitoring.

A potential solution for large‐scale eDNA sampling could be the use of robots equipped with automated collection mechanisms. The integration of robotics into environmental monitoring is becoming increasingly widespread, with robots being used for tasks such as installing sensors on trees, monitoring wildlife and assessing environmental conditions (Angelini et al. 2023; Chellapurath et al. 2023; Geckeler and Mintchev 2022; Oliveira et al. 2021). These technologies have the potential to revolutionise how we monitor ecosystems, offering precision and the ability to cover vast areas. Recently, the idea of using robots for eDNA collection has gained significant attention (Hendricks et al. 2023; Preston et al. 2024). Innovations include the use of autonomous or remotely controlled robots to collect water samples for eDNA analysis (Preston et al. 2024; Sepulveda et al. 2020) or swab vegetation for surface‐bound DNA (Aucone et al. 2023; Kirchgeorg et al. 2024). These methods can improve the efficiency and scope of eDNA sampling, enabling biodiversity monitoring across a wider range of environments, larger areas and locations that are challenging for humans to access. However, despite these advancements, no easily accessible system has yet been proposed that is both simple and cost‐effective enough to be implemented on commercially available drones. Most existing robotic systems for eDNA collection are proprietary, expensive or require specialised robotics expertise, limiting their accessibility and widespread adoption (Aucone et al. 2023). Developing a lightweight, affordable and easy‐to‐integrate eDNA collection system for commercially available drones could democratise the use of robotics in eDNA sampling, allowing researchers and conservationists to deploy these tools in a variety of settings with minimal investment. Such a system could significantly increase our ability to monitor and conserve biodiversity on a global scale. Enhanced capacity to sample large surfaces could go hand in hand with the increasing portability of sequencing machines.

One of the areas where the application of drones can greatly accelerate and simplify the sampling procedure is the monitoring of arthropods, such as insects. Some live on the surface of vegetation and, through their activity, could shed their DNA in places potentially out of reach to humans but easily accessible by drones. Insects are frequently used as excellent biodiversity indicators for the entire ecosystem (Chowdhury et al. 2023; Sharma et al. 2023) and are thus the focus of long‐term monitoring efforts. Insects play a crucial role in the food web, serving as prey for predators such as birds, and changes in insect abundance can have significant repercussions throughout the ecosystem (Cardoso et al. 2020). Traditional and most simplistic monitoring of insects includes net sweeping, which is associated with manual taxonomic identification (Kumar et al. 2022). This approach requires both extensive resources and time due to the need for trained taxonomic experts. Moreover, many insect groups are taxonomically challenging to identify and most insects remain undescribed, posing difficulties for biodiversity assessments (Stork 2018). Efforts to address this challenge have included bulk sampling using traps such as Malaise traps, followed by metabarcoding of the captured specimens (Buchner et al. 2024). While this sampling method can be effective, it has several disadvantages. For example, the trap must be set up for extended periods (up to several days), collects mainly flying invertebrates, has site‐dependent catch rates, and finally, the trapped individuals are collected dead. Moreover, the processing of the samples afterwards can be time‐consuming and labour‐intensive. An alternative approach to identification involves using insect‐specific primers for eDNA analysis to assess insect diversity over large areas. This method can offer a more efficient way to quickly monitor insect populations across extensive habitats, such as grasslands, forests or others, providing valuable data for conservation and ecological studies without the need for prolonged trapping and laborious sample processing (Svenningsen et al. 2021; Svenningsen et al. 2024; Uhler et al. 2021; Van Klink et al. 2022). Compared to some other identification or sampling methods, it is also a non‐invasive approach as it does not require the destruction of the specimens.

In this study, we aim to evaluate how a combination of novel methodologies can support the monitoring of insects associated with different types of vegetation. We used a method that combines a commercial‐off‐the‐shelf drone with eDNA sampling by vegetation swabbing and the fast reading of DNA sequences with the third‐generation sequencing of the Oxford Nanopore Technologies (ONT). This approach exploits the mobility and precision of drones to efficiently collect eDNA samples over large areas, making it a promising tool for rapid biodiversity assessments. Additionally, the employment of the ONT MinION sequencing device enables fast processing of eDNA samples and provides quicker results compared to traditional sequencing platforms like MiSeq. This method is tested in a grassland, shrubland and forest area in Switzerland, where we evaluate its effectiveness in capturing a comprehensive snapshot of local biodiversity. Specifically, we ask the following questions:

1. Can a combination of drone‐aided swabbing and Oxford nanopore sequencing recover insect species on different vegetation types and compare them meaningfully?
2. What is the sampling effort required to recover the entire insect assemblage associated with above‐ground vegetation using drone‐aided swabbing?
3. Can drone‐aided swabbing and Oxford Nanopore sequencing be used to identify the known differences in insect communities across three vegetation types: grasslands, shrubs and trees?

With the demonstration of its performance, there is a scope for the deployment of this system to ease the monitoring of these habitats.

## 2 Materials and Methods

### 2.1 Probe Design and Field Sampling With Drones

The fieldwork was carried out in Birmensdorf on a dry grassland area surrounded by shrubs and forest (47.345083°, 8.449900° close to Birmensdorf, Canton of Zürich, Switzerland). We selected a specific type of agricultural landscape, a mesic dry grassland habitat within the National Ecological Network (REN), surrounded by hedges and forest. The sampled forest was composed of deciduous species, with a dominance of oak (*Quercus* spp.) and ash ( *Fraxinus excelsior* ). The hedges were composed of various species, including ash ( *Fraxinus excelsior* ), ivy ( *Hedera helix* ), hazel ( *Corylus avellana* ), wild cherry ( *Prunus avium* ), hawthorn ( *Crataegus monogyna* ) and brambles ( *Rubus fruticosus* ).

**FIGURE 1:**
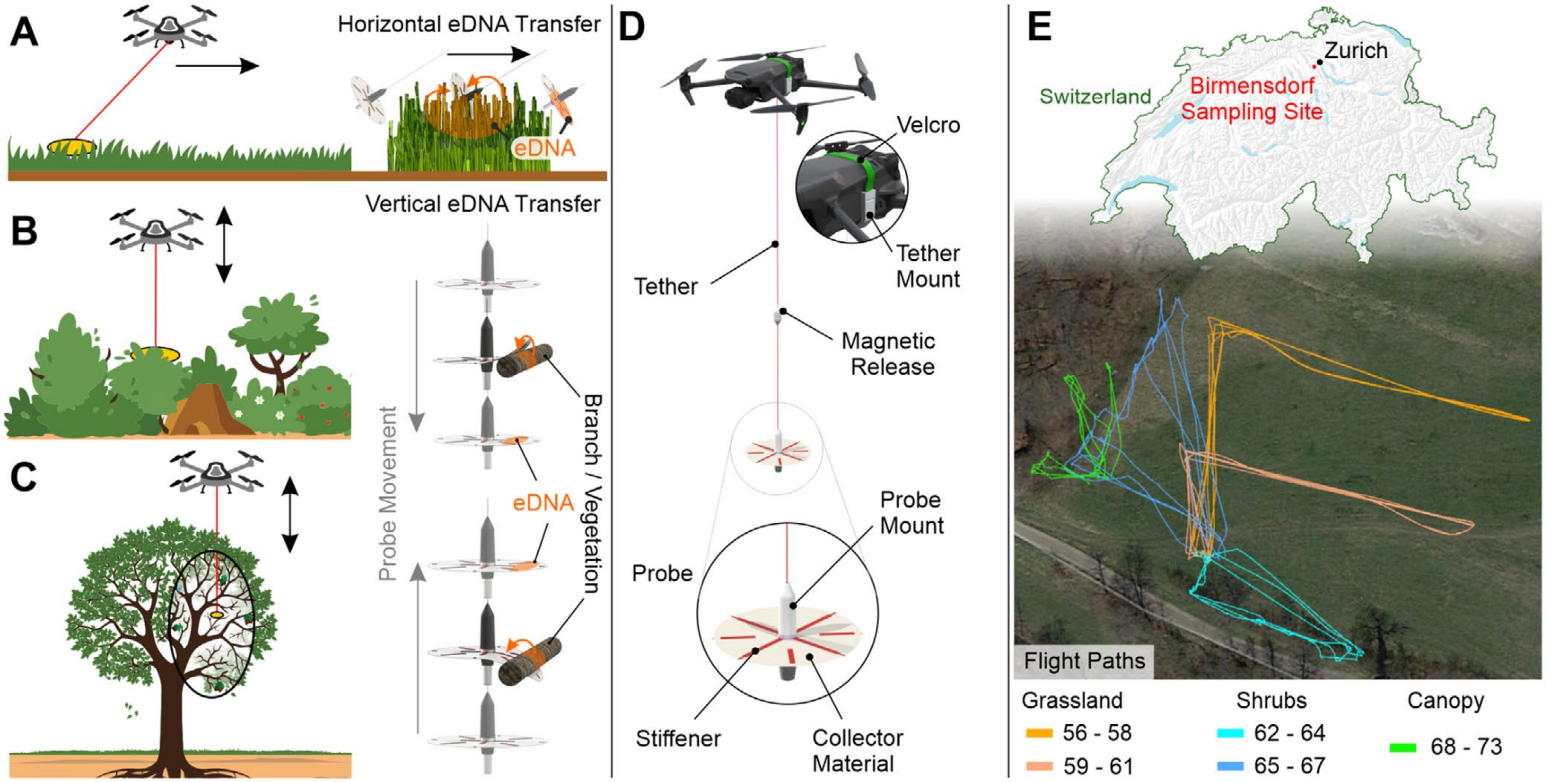
Robotic system (A) Horizontal dragging of the probe to sample grasslands, (B) vertical sampling in shrubs and (C) vertical sampling in forest canopies. (D) System overview with the velcro‐fixed tether mount on the drone, the magnetic release, and the probe with its collector material and stiffeners. (E) Satellite image of the sampling location close to Birmensdorf, Switzerland with the flight paths for the different vegetation types and sample numbers (SWA‐2023‐XX).

To sample this mosaic of habitats, we employed a DJI Mavic 3 consumer drone with a specialised probe to collect eDNA samples from various vegetation types (Figure 1A–C). The probe is attached to the drone using a four‐metre‐long tether, which is secured to a Velcro‐fixed mount, as shown in Figure 1D. In the middle of the tether, a magnetic release mechanism allows the attachment and detachment of the probe for easier handling during sampling. The release mechanism also prevents the drone from crashing if the probe or tether gets stuck in the vegetation. A moisturised circular surface swab is used as the eDNA collector material (Disko Lint‐Free Fleece Cloths). The swab is reinforced radially with four interwoven fibreglass stiffeners to retain its shape and thus facilitate the swabbing of vegetation. The probe swabs against vegetation while being dragged, or while descending and ascending along wild plants or crops. The swabbing method is selected based on the type of sampled vegetation.

In the grassland areas, the drone was programmed to autonomously fly a transect of ~120 m back and forth approximately five metres above the ground at a speed of 1 m/s, dragging the probe along the vegetation as it advanced (Figure 1A,E). This approach allowed for efficient and consistent swabbing of the grassland surface, ensuring that eDNA was collected from a broad area. We conducted three transects at two locations 50 m apart, collecting six samples to characterise insect biodiversity in the grassland area (Figure 1F). For sampling shrubs and trees, we applied a different technique. We flew the drone manually above the vegetation, adjusting its altitude to lower and raise the probe into the canopy or foliage (Figure 1B,C). This vertical movement ensured contact with different vegetation layers, capturing a representative sample of the genetic material present in these more complex structures. Considering the shrubs sampling, we performed three transects at two locations 80 m apart, collecting a total of six samples. To sample the canopy, we collected six samples at three distinct locations, as flying under the canopy was too risky. However, one sample was lost in a tree, leaving a total of five samples. This dual approach allowed us to adapt the sampling method to different vegetation types, maximising the eDNA recovery from the varied plant communities in the study area.

We followed a rigorous procedure to prevent contamination between samples during fieldwork, using single‐use probes, gloves and Falcon tubes for each sample. This procedure starts at the beginning of sampling by opening a bag containing a sterile probe, gloves and 10 mL of ultrapure water. We put on gloves and poured ultra‐pure water over the probe. Then, we attached the probe to the drone and flew it as described above. Once the drone returned and swabbing was complete, we opened a second bag containing sterile gloves and 10 mL of Longmire buffer in a 50 mL Falcon tube. We put on the new pair of gloves, detached the probe from the drone, removed the four interwoven fibreglass stiffeners, and placed the circular surface swab into the Falcon tube. Finally, we closed and labelled the tube and repeated this procedure for all samples.

### 2.2 Laboratory DNA Extraction

DNA extraction was carried out using a modified protocol from (Pont et al. 2018), optimised for eDNA extraction from Longmire buffer (Longmire et al. 1997). The extraction process took place in a dedicated eDNA laboratory designed to minimise contamination risks. The laboratory is equipped with positive air pressure, ultraviolet light treatment and frequent air renewal systems, ensuring a controlled and clean environment for eDNA handling. To further prevent contamination, rigorous decontamination procedures were implemented both before and after all sample manipulations. These procedures included surface decontamination with DNA‐destroying agents, use of sterile consumables and handling of samples in laminar flow hoods sterilised with UV light. We chose to use a Longmire buffer obtained from a modified protocol for its efficiency in preserving DNA from environmental samples. This buffer provides high‐quality DNA suitable for downstream analyses, including high‐throughput sequencing.

### 2.3 PCR, Library Preparation and Oxford Nanopore Sequencing

Eighteen swab samples (SWA‐2023‐056—SWA‐2023‐073; see Table 1 for details) were used for the DNA amplification of the target barcoding region by polymerase chain reaction (PCR). We carried out the PCR using the MtInsects‐16S primer set (Takenaka et al. 2023) amplifying about 215 bp of the mitochondrial 16S region. We prepared the PCR reactions under a laminar flow hood; the reaction volume of 25 μL contained: 12.5 μL AmpliTaq Gold 360 Master Mix (2x; Applied Biosystems, 0.75 μL for both the F and R primers (10 μM), 2.5 μL BSA (2 μg/μL), 1.25 μL DMSO (100%), 6.25 μL molecular grade H20 and 1 μL of extracted eDNA). To the eighteen swab samples, we added two negative extraction controls and one negative PCR control (all including molecular grade H_2_0 instead of DNA) to verify that there was no contamination occurring during the process. The thermocycling conditions set on the Sensoquest labcycler were: denaturation 95°C, 10 min; 40 cycles each consisting of denaturation 95°C, 30 s; annealing 55°C, 30 s; extension 72°C, 14 s; and final extension of 5 min. We verified the PCR products with electrophoresis on a 1% agarose gel, using the GelRed dye and quantified with Qubit. In total, we performed four PCR replicates for each sample that we pooled together. Then, we used 80 μL per pooled sample for a cleanup with AmpureBeads using a 1X ratio and standard protocol. The library was prepared according to the Ligation sequencing amplicons—Native Barcoding Kit 24 V14 (SQK‐NBD114.24) protocol (Version: NBA_9168_v114_revM_15Sep2022) from Oxford Nanopore Technologies. The following adaptations to the protocol were applied: we used BSA 20 mg/mL instead of the 50 mg/mL recommended, and we used a 1X ratio of AmpureBeads throughout all the cleanup steps to retain the fragments of our desired length. We diluted the samples to equimolarity after the End‐prep reaction. Finally, we manually terminated the MinION sequencing run after 21 h 45 min. By employing the barcoding strategy, we were able to sequence multiple distinct samples in parallel on a single flow cell and thus maximise the sequencing capacity, while maintaining the ability to distinguish between samples during downstream bioinformatics analysis.

**TABLE 1:** Detailed metadata associated with the field sampling (Sample SWA_2023_0056‐SWA_2023_0073). This table provides comprehensive metadata for the sample collected in Birmensdorf on a dry grassland area surrounded by shrubs and forest, including information on the Minion code.

Filter ID	Minion_code	Start time	Duration	Sample type	Preservation	Replicate	Lat_st	Lon_st	Lat_end	Lon_end	Location name	Habitat	Ecosystem	Comment
SWA_2023_0073	SQK.NBD114.24_barcode18	30.11.2023 08:33	0	SWAB	Longmire buffer	0	47.372515	8.542192	NA	NA	Wüeribach	NA	NA	Blank test
SWA_2023_0072	SQK.NBD114.24_barcode17	06.10.2023 16:19	5	SWAB	Longmire buffer	2	47.345143	8.449757	47.345349	8.449959	Wüeribach	Forest	TEMPERATE_BROADLEAF_MIXED_FOREST	NA
SWA_2023_0071	SQK.NBD114.24_barcode16	06.10.2023 15:42	5	SWAB	Longmire buffer	1	47.345143	8.449757	47.345349	8.449959	Wüeribach	Forest	TEMPERATE_BROADLEAF_MIXED_FOREST	NA
SWA_2023_0070	SQK.NBD114.24_barcode15	06.10.2023 15:36	5	SWAB	Longmire buffer	3	47.345143	8.449757	47.345349	8.449959	Wüeribach	Forest	TEMPERATE_BROADLEAF_MIXED_FOREST	NA
SWA_2023_0069	SQK.NBD114.24_barcode14	06.10.2023 13:25	5	SWAB	Longmire buffer	2	47.345143	8.449757	47.345349	8.449959	Wüeribach	Forest	TEMPERATE_BROADLEAF_MIXED_FOREST	NA
SWA_2023_0068	SQK.NBD114.24_barcode13	06.10.2023 15:20	5	SWAB	Longmire buffer	1	47.345143	8.449757	47.345349	8.449959	Wüeribach	Forest	TEMPERATE_BROADLEAF_MIXED_FOREST	NA
SWA_2023_0067	SQK.NBD114.24_barcode12	06.10.2023 15:10	5	SWAB	Longmire buffer	3	47.344924	8.450189	47.344808	8.450656	Wüeribach	Shrubs	TEMPERATE_GRASSLANDS_SAVANNAS_SHRUBLANDS	NA
SWA_2023_0066	SQK.NBD114.24_barcode11	06.10.2023 14:50	5	SWAB	Longmire buffer	2	47.344924	8.450189	47.344808	8.450656	Wüeribach	Shrubs	TEMPERATE_GRASSLANDS_SAVANNAS_SHRUBLANDS	NA
SWA_2023_0065	SQK.NBD114.24_barcode10	06.10.2023 14:40	5	SWAB	Longmire buffer	1	47.344924	8.450189	47.344808	8.450656	Wüeribach	Shrubs	TEMPERATE_GRASSLANDS_SAVANNAS_SHRUBLANDS	NA
SWA_2023_0064	SQK.NBD114.24_barcode09	06.10.2023 13:15	5	SWAB	Longmire buffer	3	47.345154	8.449847	47.345467	8.450148	Wüeribach	Shrubs	TEMPERATE_GRASSLANDS_SAVANNAS_SHRUBLANDS	NA
SWA_2023_0063	SQK.NBD114.24_barcode08	06.10.2023 13:10	5	SWAB	Longmire buffer	2	47.345154	8.449847	47.345467	8.450148	Wüeribach	Shrubs	TEMPERATE_GRASSLANDS_SAVANNAS_SHRUBLANDS	NA
SWA_2023_0062	SQK.NBD114.24_barcode07	06.10.2023 12:30	5	SWAB	Longmire buffer	1	47.345154	8.449847	47.345467	8.450148	Wüeribach	Shrubs	TEMPERATE_GRASSLANDS_SAVANNAS_SHRUBLANDS	NA
SWA_2023_0061	SQK.NBD114.24_barcode06	06.10.2023 12:15	5	SWAB	Longmire buffer	3	47.345418	8.450269	47.345242	8.451115	Wüeribach	Grassland	TEMPERATE_GRASSLANDS_SAVANNAS_SHRUBLANDS	NA
SWA_2023_0060	SQK.NBD114.24_barcode05	06.10.2023 12:10	5	SWAB	Longmire buffer	2	47.345418	8.450269	47.345242	8.451115	Wüeribach	Grassland	TEMPERATE_GRASSLANDS_SAVANNAS_SHRUBLANDS	NA
SWA_2023_0059	SQK.NBD114.24_barcode04	06.10.2023 12:05	5	SWAB	Longmire buffer	1	47.345418	8.450269	47.345242	8.451115	Wüeribach	Grassland	TEMPERATE_GRASSLANDS_SAVANNAS_SHRUBLANDS	NA
SWA_2023_0058	SQK.NBD114.24_barcode03	06.10.2023 11:57	5	SWAB	Longmire buffer	3	47.345187	8.450176	47.345051	8.450965	Wüeribach	Grassland	TEMPERATE_GRASSLANDS_SAVANNAS_SHRUBLANDS	NA
SWA_2023_0057	SQK.NBD114.24_barcode02	06.10.2023 11:50	5	SWAB	Longmire buffer	2	47.345187	8.450176	47.345051	8.450965	Wüeribach	Grassland	TEMPERATE_GRASSLANDS_SAVANNAS_SHRUBLANDS	NA
SWA_2023_0056	SQK.NBD114.24_barcode01	06.10.2023 13:25	5	SWAB	Longmire buffer	1	47.345187	8.450176	47.345051	8.450965	Wüeribach	Grassland	TEMPERATE_GRASSLANDS_SAVANNAS_SHRUBLANDS	NA

### 2.4 Bioinformatic Analyses

We conducted the eDNA analysis using our custom pipeline that interfaces together multiple software tools to ensure robust taxonomic assignment and efficient filtering of hits from the reference database (the pipeline is available here: https://gitlab.ethz.ch/ele‐public/edna‐minion). The pipeline adds an extra layer of parallelization for improved performance and allows full parameterization of each step via a single configuration file to ensure reproducibility. We first basecalled the sequencing outputs using Dorado (v0.7.2) with the high‐accuracy model (dna_r10.4.1_e8.2_400bps_sup@v5.0.0), applying a minimum average Q‐score threshold of 12 to retain high‐confidence reads. Through Dorado's demultiplexing module, we assigned reads to their respective samples, barcoded with the SQK‐NBD114‐24 standard kit. We performed the amplicon sorting using the *Amplicon_sorter* tool (Vierstraete and Braeckman 2022), which groups sequences by similarity. We excluded reads shorter than 115 bases or longer than 315 bases, randomly selecting batches of 1000 reads for comparison. We clustered the reads together with 97% similarity and merged clusters if their consensus sequences matched at 98%. Taxonomic assignment of consensus sequences was performed using blastn (v2.15.0+; Camacho et al. 2009) against the MIDORI2 16S reference database (vGB261; Leray et al. 2022). We applied an e‐value threshold of 1e‐3 and required a minimum of 80% identity and coverage, retaining the top 20 hits for each consensus sequence when available. Finally, we applied a custom Python script to refine the taxonomic assignments. Species‐level assignments were accepted for sequences with ≥ 98% identity, genus‐level with ≥ 96% and family‐level with ≥ 90% (Polanco et al. 2022). In cases where multiple hits shared the highest bit‐score, we retained only the common taxonomic levels among these top‐ranked hits.

### 2.5 Statistical Analyses

To determine the appropriate number of samples for our sampling and to ensure a sufficient probability of detecting an effect of interest at a given significance level, we performed a power calculation for a balanced one‐way analysis of variance (ANOVA). To do so, we defined 4 parameters, the effect size, the significance level (0.05), the number of groups (grassland, forest and shrubs) as well as the statistical power (0.8). As we expected a large effect size, meaning the differences between group means are expected to be substantial relative to the within‐group variability, we set‐up this parameter to 0.75. The grassland samples will be more similar to each other than samples taken in the forest and shrubs. By accounting for these parameter values and assuming this effect size, the required sample size per group was six. To conduct this analysis, we used the *pwr.anova.test* function from the *pwr* R package (Champely 2020).

We performed a taxa accumulation analysis to assess the number of samples required to capture the entire insect diversity across the three considered habitat types: Grassland, Shrubs and Forest. We used the specaccum and fitspecaccum functions from the R package vegan (Oksanen et al. 2025), which calculate the expected taxa accumulation curve using a sample‐based rarefaction method and then fit nonlinear accumulation models. We tested three different models (Lomolino, asymptotic and logistic) and identified the best fit by selecting the model with the lowest Akaike Information Criterion (AIC) for each habitat. Based on the AIC, we then fitted the best model to the data and estimated the asymptote value for each type of habitat.

To assess and compare the insect diversity among different types of vegetation swabbed, we computed‐diversity metrics, specifically the taxa richness and the hill number (q1) as a measure of evenness (Chao et al. 2014), for each vegetation type and each sample. To compare diversity levels across vegetation types, we applied a poisson general linear model between taxa richness and habitat types and a linear model between evenness and habitat types. For the two models, we checked the homogeneity of variance of the explained variable in each habitat and the model residuals for normality by applying both a Levene and a Shapiro tests (Royston 1982). For the poisson general linear model we calculated the estimated marginal means (EMMs) on the log‐transformed scale for each habitat type and we used the Tukey method for multiple comparisons to test for pairwise differences between habitats. Considering the evenness, we performed an analysis of variance followed by a post hoc analysis Tukey's ‘Honestly Significant Difference’ test (Miller Jr. 2011). We conducted aTukey HSD post hoc test to examine pairwise differences in taxa richness across habitats (forest, grassland and shrubs) at a 95% confidence level. We tested whether taxa richness and evenness differ across habitat types.

For assessing *β‐*diversity, we created a presence–absence matrix based on the taxa detected, and we calculated the pairwise Jaccard dissimilarity along with its two additive components, the taxa turnover (*β*_*jtu*_) and the nestedness (*β*_*jnes*_) between sites (Anderson et al. 2011; *β*_*jac*_) by using the function ‘beta. pair’ of the R package ‘Betapart’ (Baselga and Orme 2012). We only represent beta turnover because it was the major component of beta diversity in our case. We then used a Permutational Multivariate Analysis of Variance (PERMANOVA) on both the *β*_*jac*_ and *β*_*jtu*_ to assess the effects of habitat type, replicate and time (hour of the day when the sample was taken) on differences in insect composition. To visualise and analyse the compositional differences among the vegetation types, we performed a Principal Coordinates Analysis (PCoA) based on both *β*_*jac*_ and *β*_*jtu*_ dissimilarity matrices. This ordination method allowed us to identify patterns in insect community composition and assess how distinct or similar the communities were across different vegetation types. The results were interpreted in conjunction with the statistical significance from PERMANOVA, providing a comprehensive understanding of the insect diversity and community structure in relation to the vegetation types swabbed.

## 3 Results

### 3.1 Deployment of the Probe

The robotic sampling system used for collecting eDNA from vegetation was designed with simplicity and efficiency in mind. Mounting the system onto the DJI drone was straightforward, requiring minimal effort and no specialised tools. The components were engineered for compatibility, allowing for a seamless integration with the drone's existing hardware. The entire setup operated as a plug‐and‐play system, meaning that once mounted, the drone and sampling probe were ready for immediate use without any complex calibration or additional configuration. This ease of use greatly enhanced the efficiency of field operations, enabling quick deployment and consistent sampling across different vegetation types.

### 3.2 Bioinformatic Analyses

Sequencing produced a total of 5.16 gigabases from 13.35 million reads, with an estimated N50 of 364 bases. After basecalling and applying a minimum quality score filter of 12, we retained 438,026 reads. Demultiplexing assigned 418,838 reads to the 18 samples, while 400 reads were linked to three unused barcodes from the kit probably associated with amplification errors, 3258 reads were attributed to the blank sample, and 15,530 reads remained unassigned to any barcode. The reads from the 18 samples were clustered into 371 groups. Of the 418,838 reads, 32,204 were outside the expected length range, and 5647 were not assigned to any group. Reads within each group were merged into 371 consensus sequences, of which 316 were identified during the taxonomic assignment phase, resulting in 229 unique sequences associated with 86 taxa. After excluding non‐insect taxa (Primates—i.e. human; *Capreolus capreolus—*roe deer; Metazoa—too general), we obtained 76 taxa that we analysed further. Among these taxa, we retained also the representatives of Collembola that were previously recognised as insects and are now considered a related lineage to insects within the phylum Hexapoda.

### 3.3 Taxa Detected

The DNA sequencing of swabbed vegetation samples yielded a diverse assemblage of arthropod species, highlighting the biodiversity within the sampled ecosystems. In the 18 samples, we detected 76 insect (hexapode) taxa. Out of this, 35 were detected to species, 10 to genus, 22 to family and 9 to order level. These taxa were detected across various taxonomic orders and families. Among Coleoptera (beetles), notable species included *Acalles dubius* (family Curculionidae) and *Pyrrhidium sanguineum* (family Cerambycidae). Diptera (flies) were represented by taxa like *Culex* mosquitoes and *Corynoptera* species (family Sciaridae). In Hymenoptera (ants, bees, wasps), *Aphelinus chaonia* (family Aphelinidae) and *Vespula vulgaris* (the common wasp) were detected. Hemiptera (true bugs) included the invasive *Halyomorpha halys* (family Pentatomidae). Lepidoptera (butterflies and moths) featured *Laothoe populi* and *Macroglossum stellatarum* (family Sphingidae). Psocodea (booklice) included *Graphopsocus cruciatus* and *Trichadenotecnum sexpunctatum*. In Orthoptera (grasshoppers and crickets), *Tettigonia viridissima* was detected. The full list of detected taxa is presented in Table 2.

**TABLE 2:** Taxa detected in different habitats indicating their number of occurrences in grassland, shrubs, and forest and the number of reads obtained per each taxon in each habitat.

Taxon	Family	Order	Nb—Grassland	Nb—Shrubs	Nb—Forest	Reads grassland	Reads shrubs	Reads forest
*Acalles dubius*	Curculionidae	Coleoptera	1	0	0	39	0	0
Acrididae	Acrididae	Orthoptera	1	0	0	1035	0	0
*Agrilus olivicolor*	Buprestidae	Coleoptera	0	1	0	0	148	0
*Aphelinus chaonia*	Aphelinidae	Hymenoptera	0	1	0	0	18,564	0
Apionidae	Apionidae	Coleoptera	1	0	0	352	0	0
*Athalia*	Athaliidae	Hymenoptera	2	0	0	497	0	0
Athaliidae	Athaliidae	Hymenoptera	1	0	0	52	0	0
*Baetis*	Baetidae	Ephemeroptera	1	0	0	43	0	0
Braconidae	Braconidae	Hymenoptera	1	4	1	15	363	86
Caeciliusidae	Caeciliusidae	Psocodea	1	1	2	44	108	37
Carabidae	Carabidae	Coleoptera	0	1	0	0	413	0
Cerambycidae	Cerambycidae	Coleoptera	0	1	0	0	1437	0
*Chaetocnema hortensis*	Chrysomelidae	Coleoptera	3	1	0	272	411	0
Chironomidae	Chironomidae	Diptera	0	0	1	0	0	323
Chrysomelidae	Chrysomelidae	Coleoptera	1	1	0	120	979	0
Cicadellidae	Cicadellidae	Hemiptera	3	0	0	210	0	0
Coleoptera	—	Coleoptera	3	3	3	2108	708	10,218
*Corynoptera perpusilla*	Sciaridae	Diptera	2	0	0	5088	0	0
*Corynoptera tetrachaeta*	Sciaridae	Diptera	1	0	0	232	0	0
*Crepidodera aurat*a	Chrysomelidae	Coleoptera	0	1	0	0	171	0
*Culex*	Culicidae	Diptera	1	0	0	141	0	0
*Curculio glandium*	Curculionidae	Coleoptera	0	0	1	0	0	241
Curculionidae	Curculionidae	Coleoptera	1	2	1	84	1167	268
*Dasytes*	Melyridae	Coleoptera	0	1	1	0	242	20,128
Diptera	—	Diptera	2	0	0	9854	0	0
*Ecdyonurus*	Heptageniidae	Ephemeroptera	1	0	0	64	0	0
Ectopsocidae	Ectopsocidae	Psocodea	1	2	3	10	170	8517
*Ectopsocus*	Ectopsocidae	Psocodea	1	1	0	341	10,814	0
*Ectopsocus meridionalis*	Ectopsocidae	Psocodea	3	3	3	921	8559	392
Entomobryidae	Entomobryidae	Entomobryomorpha	5	0	0	267	0	0
Entomobryomorpha	—	Entomobryomorpha	6	0	2	62,586	0	535
*Forficula auricularia*	Forficulidae	Dermaptera	3	0	0	4912	0	0
*Galeruca luctuosa*	Chrysomelidae	Coleoptera	0	1	0	0	61	0
*Graphopsocus*	Psocidae	Psocodea	0	0	1	0	0	12
*Graphopsocus cruciatus*	Psocidae	Psocodea	0	2	4	0	26,951	11,633
*Halyomorpha halys*	Pentatomidae	Hemiptera	1	0	0	42	0	0
Hemiptera	—	Hemiptera	2	0	3	7715	0	43,051
Heptageniidae	Heptageniidae	Ephemeroptera	1	0	0	2113	0	0
Hymenoptera	—	Hymenoptera	2	3	0	151	14,413	0
*Ischnopterapion virens*	Apionidae	Coleoptera	1	0	0	186	0	0
*Issus kabylicus*	Issidae	Hemiptera	0	1	0	0	40	0
*Laothoe*	Sphingidae	Lepidoptera	0	1	0	0	1285	0
*Laothoe populi*	Sphingidae	Lepidoptera	0	1	0	0	62	0
Lepidoptera	—	Lepidoptera	1	0	0	18	0	0
*Leptophyes punctatissima*	Tettigoniidae	Orthoptera	0	0	1	0	0	125
*Leuctra fusca*	Leuctridae	Plecoptera	2	0	0	3651	0	0
*Liorhyssus hyalinus*	Rhopalidae	Hemiptera	1	0	0	24	0	0
*Longitarsus luridus*	Chrysomelidae	Coleoptera	1	0	0	26	0	0
*Longitarsus pratensis*	Chrysomelidae	Coleoptera	3	0	0	143	0	0
*Longitarsus salviae*	Chrysomelidae	Coleoptera	3	0	0	210	0	0
*Macroglossum stellatarum*	Sphingidae	Lepidoptera	1	0	0	33	0	0
Melyridae	Melyridae	Coleoptera	0	0	1	0	0	86
Neuroptera	—	Neuroptera	1	0	0	1555	0	0
*Nipponomeconema*	Tettigoniidae	Orthoptera	1	0	0	109	0	0
*Noctua pronuba*	Noctuidae	Lepidoptera	0	1	0	0	28	0
Noctuidae	Noctuidae	Lepidoptera	0	1	0	0	565	0
*Ocypus olens*	Staphylinidae	Coleoptera	1	0	0	108	0	0
*Oscinella pusilla*	Chloropidae	Diptera	1	0	0	31	0	0
Peripsocidae	Peripsocidae	Psocodea	0	0	1	0	0	1928
*Peripsocus subfasciatus*	Peripsocidae	Psocodea	0	2	0	0	245	0
Psocidae	Psocidae	Psocodea	0	0	1	0	0	104
Psocodea (Psocoptera)		Psocodea	0	2	1	0	1630	2251
Psychodidae	Psychodidae	Diptera	2	3	1	105	491	11
*Pyrausta despicata*	Crambidae	Lepidoptera	1	0	0	40	0	0
*Pyrrhidium sanguineum*	Cerambycidae	Coleoptera	2	0	0	113	0	0
*Rhopalosiphum padi*	Aphididae	Hemiptera	0	1	0	0	39	0
*Rhynchaenus quercus*	Curculionidae	Coleoptera	0	0	1	0	0	98
Sciaridae	Sciaridae	Diptera	2	1	1	72	16	221
Sphingidae	Sphingidae	Lepidoptera	0	1	0	0	86	0
*Tettigonia viridissima*	Tettigoniidae	Orthoptera	1	0	0	21	0	0
Thripidae	Thripidae	Thysanoptera	4	0	0	833	0	0
Thysanoptera		Thysanoptera	2	0	0	3112	0	0
*Trichadenotecnum sexpunctatum*	Psocidae	Psocodea	0	2	0	0	518	0
*Valenzuela*	Psocidae	Psocodea	1	1	0	11	199	0
*Vespula vulgaris*	Vespidae	Hymenoptera	0	1	0	0	13	0
*Xyleborus monographus*	Curculionidae	Coleoptera	0	0	1	0	0	603

### 3.4 Taxa Accumulation Curve

**FIGURE 2:**
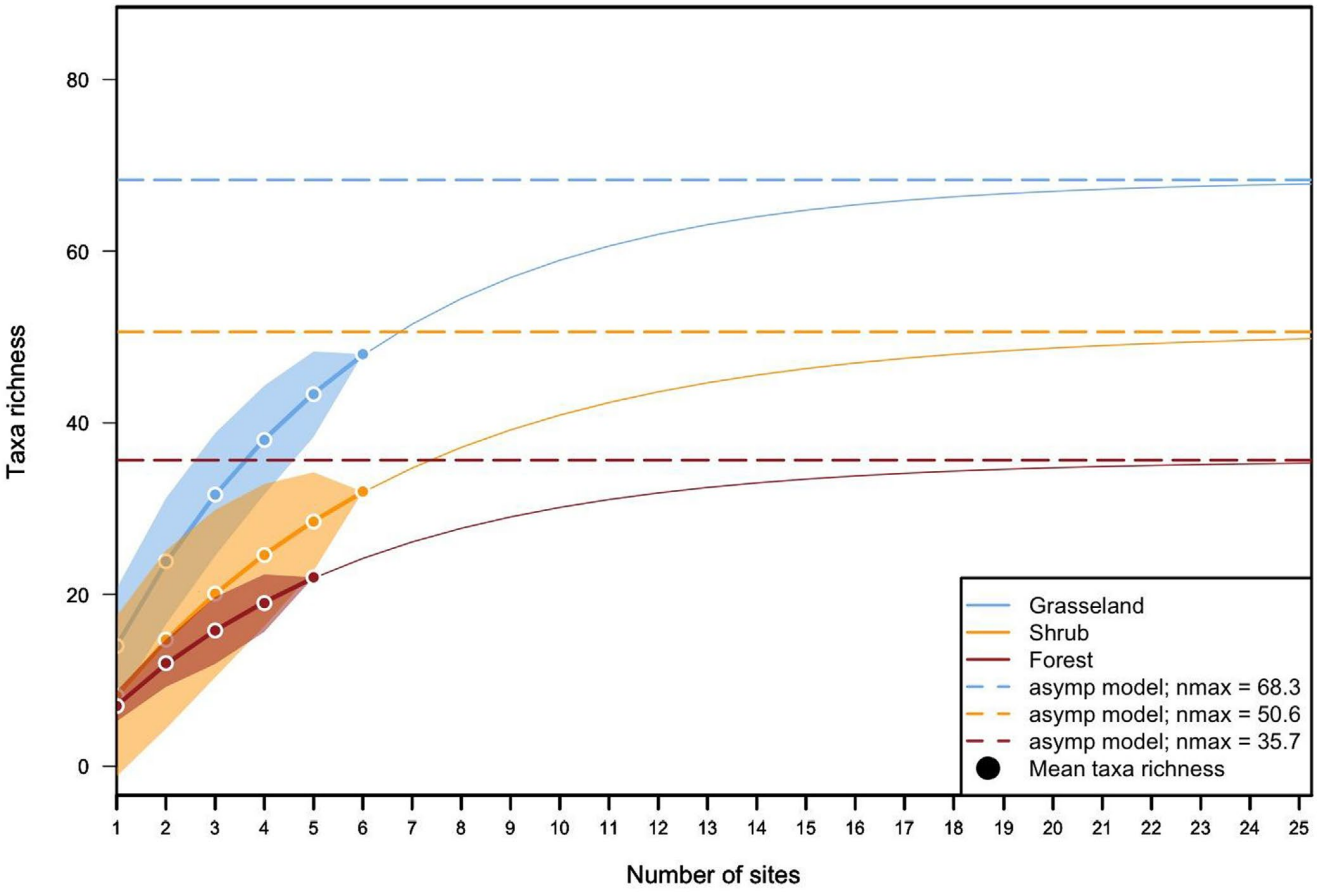
Taxa accumulation curves depicting taxa richness across the three sampled habitat types: Grassland (blue line), shrubs (orange line), and forest (red line). The dashed lines represent asymptotic models for each habitat, indicating the estimated maximum number of taxa expected: Grassland (nmax = 68.3), shrubs (*n*_max_ = 50.6), and forest (*n*_max_ = 35.7). The solid circles indicate the mean taxa richness observed for each habitat at the corresponding number of sites sampled. These curves illustrate the biodiversity patterns across different ecosystems and highlight the distinct species composition associated with each habitat type. The coloured envelope represents the standard deviation around the mean taxa richness.

The taxa accumulation curves from eDNA samples across three habitats—Grassland, Shrub and Forest—show distinct patterns of taxa richness as the number of sites increases (Figure 2). Grassland consistently demonstrated the highest taxa richness, with an asymptotic model predicting a maximum taxa richness of 69.3. Shrub habitats followed, with a predicted maximum of 50.6 taxa, while forest habitats showed the lowest taxa richness, with an asymptotic maximum of 35.7. Considering the three types of habitats, the model with the lower AIC was the asymtotic model. The curves for each habitat approach asymptotes, suggesting that additional sampling would likely yield fewer new taxa, especially for the forest sites, where taxa richness levels off earlier. The mean taxa richness per habitat reflects this gradient, with grassland sites hosting the most diverse assemblages, followed by shrubs, and forests showing the lowest diversity. These results highlight the varying capacity of different habitats to harbour diverse species assemblages, as captured through eDNA sampling.

### 3.5 Diversity and Composition Analyses

**FIGURE 3:**
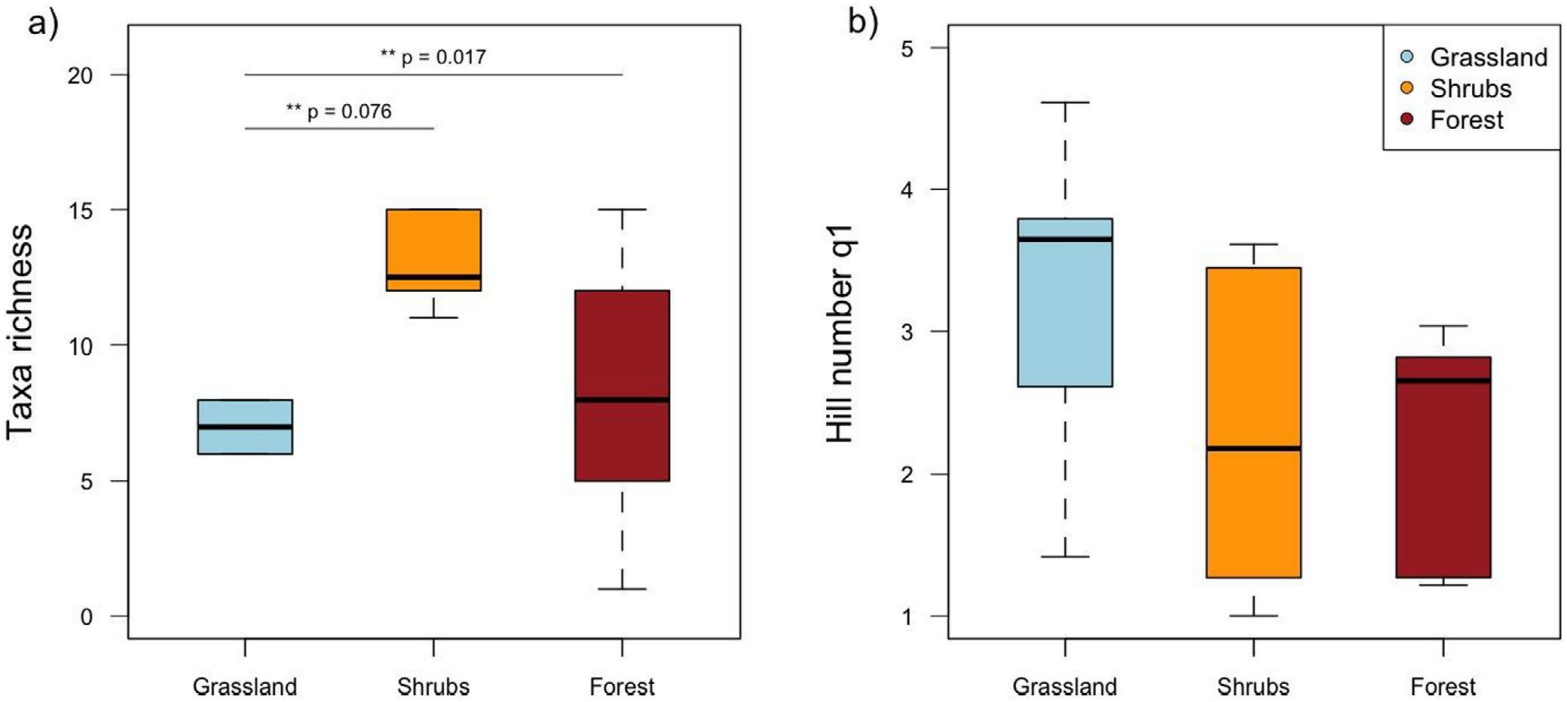
Number of taxa recovered across the different types of habitats sampled. These figures show (a) A boxplot of the taxa richness per sample in grasslands, shrubs, and forests, and (b) A box plot of the evenness calculated as q1 Hill number of reads within samples for the same three habitat types. (**indicates statistical significance at the 0.05 level for the results of a Tukey HSD post hoc test).

We found that insect taxa richness varies significantly between habitats (Figure 3a) with grassland showing a higher taxa richness compared to forest (Tukey contrast; estimate = −0.693, 95% CI [−1.17, −0.222], *p* = 0.017) and shrubs (Tukey contrast; estimate = 0.539, 95% CI [0.12, 0.96], *p* = 0.076). No significant difference in insect taxa richness was found between shrubs and forest habitats (Tukey contrast; estimate = −0.154, 95% CI [−0.67, 0.37], *p* = 0.77). Moreover, the data used to conduct the previous test meet the assumption of homogeneity of variance (Levene's test; *F* = 2.86, *p* = 0.09) and the residuals from the linear model used to conduct the analysis of variance (predicting species richness based on habitat) were normally distributed (Shapiro test, *W* = 0.94, *p* = 0.337).

We did not find a significant difference in the evenness of reads between grassland and forest habitats (Tukey HSD post hoc; mean difference = 1.09, 95% CI [−0.57, 2.75], *p* = 0.234). Similarly, the difference in evenness between shrubs and forest habitats was not significant (Tukey HSD post hoc; mean difference = 0.08, 95% CI [−1.58, 1.74], *p* = 0.964). Moreover, the data used to conduct the previous test met the assumption of homogeneity of variance (Levene's test; *F* = 0.131, *p* = 0.87), and the residuals from the linear model used to conduct the analysis of variance were normally distributed (Shapiro test, *W* = 0.94, *p* = 0.322). To conclude, we did not detect significant differences in the evenness of reads among the different habitat types, suggesting a similar distribution of insect taxa across grassland, forest and shrub habitats.

**FIGURE 4:**
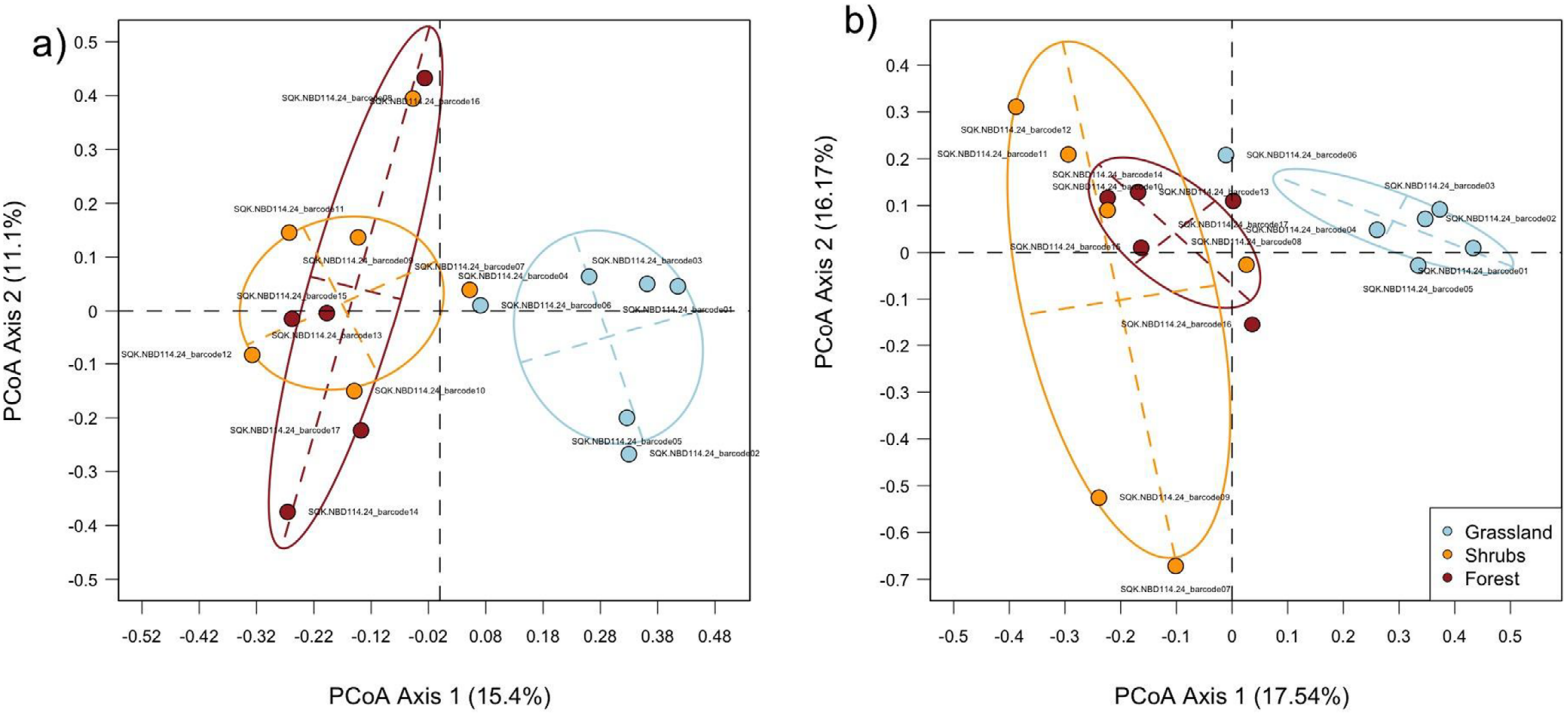
Ordination of the swabbing samples obtained from a PCoA analysis conducted on the community matrix. Shown are the results (a) with a PCoA based on Jaccard distances and (b) with a PCoA based only on the taxa turnover components. Results show major compositional differences between grasslands and woody vegetation and less difference between shrubs and trees.

In terms of taxa composition, we found that the insect composition was very different in grassland samples compared to those of forest or shrubs, which were more similar. The results of the PERMANOVA on *β*_*jac*_ diversity indicate that habitat type significantly influences insect community composition (*F* = 1.42, *p* = 0.001; Figure 4a), explaining 21.67% of the variance. In contrast, replicate (*F* = 0.898, *p* = 0.681) and sampling time (*F* = 0.9538, *p* = 0.559) do not have a meaningful impact. The variation in insect composition between grassland and the other habitat types is primarily driven by taxa turnover (mean βjac = 0.901 ± 0.09; mean βjtu = 0.85 ± 0.15). The PERMANOVA results on *β*_*jtu*_ diversity further indicate that habitat explains a significant portion of insect *β*‐diversity variation (*R* ^2^ = 0.23, *F* = 2.112, *p* = 0.001; Figure 4b), while replicate (*R* ^2^ = 0.046, *F* = 0.848, *p* = 0.643) and time (*R* ^2^ = 0.069, *F* = 1.262, *p* = 0.227) do not significantly contribute to the explained variation. Overall, 65.45% of the variation remains unexplained, suggesting that additional factors may influence beta diversity.

Some insect species were found exclusively in grasslands, demonstrating the unique community composition of this habitat. These include *Acalles dubius* (family Curculionidae), a small weevil commonly associated with dry, open habitats, Acrididae (family Acrididae), a group of grasshoppers known for their adaptations to grassy environments, and *Forficula auricularia* (family Forficulidae), the common earwig, which thrives in the ground litter and low vegetation typical of grasslands. Additionally, species from the Chrysomelidae family, such as *Chaetocnema hortensis* and *Longitarsus pratensis* , were prominent in grassland habitats, highlighting the specialisation of these leaf beetles in herbaceous plant communities. In contrast, several species were exclusively associated with shrub and forest environments, showcasing the importance of more complex vegetation structures in supporting different insect communities. Notable among these are *Agrilus olivicolor* (family Buprestidae), a wood‐boring beetle known for inhabiting shrubs and trees, and *Aphelinus chaonia* (family Aphelinidae), a parasitoid wasp that preys on aphids and is more commonly found in dense, woody vegetation. Other shrub and forest specialists include Carabidae (ground beetles) such as *Dasytes* (family Melyridae) and Cerambycidae (longhorn beetles), both of which are dependent on the diverse plant structures and microhabitats provided by woody environments. This contrast in species composition between grasslands and shrub/forest habitats underscores the habitat‐specific ecological roles these species play.

## 4 Discussion

The integration of drones aided sampling and eDNA offers a promising advancement in biodiversity monitoring, addressing some limitations of traditional methods while providing reliable results in our case study. Overall, the insect composition reveals well‐structured communities among the different vegetation types sampled (Figure 3). The eDNA metabarcoding approach was able to provide an overview of a diversity of functional groups within the sampled vegetation, comprising herbivores, predators, parasitoids and decomposers, which together contribute to the ecological dynamics of the grassland and shrubland environments (Debinski 2023). In terms of the methodology, our study demonstrates how the integration of various cutting‐edge technologies can be integrated to develop a novel tool for biodiversity monitoring, specifically targeting the DNA of insects. By combining drone‐based sampling, eDNA analysis, and nanopore sequencing, we have created a system that allows for the efficient detection of insect biodiversity directly from plant surfaces and which can be applied to biodiversity assessment of natural vegetation but also applied to the agricultural field. This study highlights the potential of using plant surfaces as a non‐invasive and effective source of eDNA for monitoring insect populations.

Insects that live on the vegetation are expected to leave traces of DNA that can be recovered by leaf swabs (Thomsen and Sigsgaard 2019). Consistent with this observation, our results revealed a diversity of insect DNA present on the leaves of various plant species. As Insects spend considerable time feeding on leaves, they leave traces of their DNA through excrement, saliva and other biological materials (Macher et al. 2023; Valentin et al. 2020). Here, we used a device that lightly swabs the vegetation, but we still lack a good understanding of the level of DNA concentration on the plants, which could vary depending on the insect activity (Kudoh et al. 2020). Understanding the distribution and concentration of insect DNA on different parts of plants could significantly enhance our ability to collect eDNA more efficiently and improve the probe used here. As this field of research is still in its infancy, with the first robot‐aided eDNA collection only dating less than five years (Yamahara et al. 2019), there is tremendous potential for refining and optimising our methods to improve accuracy and expand the applicability of this approach. By further exploring the interactions between insects and plants, we can develop more targeted and effective strategies for eDNA collection, paving the way for advanced biodiversity monitoring techniques that can be applied across a wide range of ecosystems.

Among the taxa we detected, 13 are rather rare in Switzerland or at some localities (*Acalles dubius*, *Agrilus olivicolor*, *Aphelinus chaonia*, *Corynoptera perpusilla*, *Corynoptera tetrachaeta*, *Crepidodera aurata*, *Galeruca luctuosa*, *Issus kabylicus*, *Liorhyssus hyalinus* , *Longitarsus salviae*, *Megalothorax willemi*, *Pyrausta despicata*, *Xyleborus monographus*). Four are wood pests (*Agrilus olivicolor*, Cerambycidae, *Xyleborus monographus*, *Rhynchaenus quercus*) and thirteen are significant or potential crop pests (Acrididae, *Agrilus olivicolor*, *Chortensis*, *Corynoptera*, *Corynoptera tetrachae*, *Curculio glandium*, Curculionidae, *Halyomorpha halys*, *Leptophyes punctatissima*, *Longitarsus salviae*, *Rhopalosiphum padi* , Thripidae, *Tettigonia viridissima*). This demonstrates that our method is capable of detecting rare species and significant or potential wood or crop pests. As such, it shows great potential for use in insect species conservation management and for preventing losses in agriculture and forestry through the early detection of harmful or invasive taxa in specific vegetation areas. Furthermore, it can be potentially capable of detecting taxa that are otherwise difficult to sample by traditional methods (including microscopic taxa like Collembola). Nevertheless, we need to underline that the assignment accuracy and reliability of the method are currently difficult to evaluate and standardise. For a correct species determination, the choice of barcode is crucial, as sister species may have a similar specific barcode sequence and are then difficult to be precisely distinguished. Similarly, some of the rare species may still be absent from barcode databases or be represented by fewer sequences than common species or frequently occurring pests.

Different vegetation structures are associated with different composition and diversity of insect assemblages (Mota et al. 2023; Tobisch et al. 2023). Grasslands are typically very rich in plant species (Grimaldi and Engel 2005; Petermann and Buzhdygan 2021) and given that many insects are herbivores, often specialists (Grimaldi and Engel 2005; Petermann and Buzhdygan 2021), the higher diversity of insects reflects the diversity of food sources. In contrast, shrub and forest layers were composed of only a few plant species (as the forest and shrub area were managed and were thus less diverse than an old‐growth forest). Thus, while offering more structured habitats, they do not provide the same diversity of food sources as grassland (Borer and Risch 2024). This contrasts with the high diversity of the mesic dry grasslands that we sampled here, which is associated with high insect diversity (Debinski 2023). This study focuses on a specific type of agricultural landscape: managed lowland grasslands, including the surrounding shrubs and forest edges, which are generally wide areas and time‐consuming to sample. While our method is applied here to dry grasslands and adjacent shrub and tree vegetation, its potential extends to a wide range of agricultural practices. Currently, there is no standardised method for assessing the impact of different agricultural practices on aboveground biodiversity, which is also hindered by the fact that agricultural practices and crop type are not uniformly reported in most countries (Damiani et al. 2023). Most biodiversity assessments are soil‐based, leaving a gap in understanding how practices like the adoption of biological agriculture, particularly changes in pesticide use, benefit organisms that live above the soil.

The MinION technology has demonstrated a promising ability to accurately read DNA sequences, making it a valuable tool for various genomic applications (Zhang et al. 2024). Here, we utilised a primer set originally designed for generating short reads on Illumina platforms to target insects. While these primers are optimised for short‐read sequencing, the MinION platform has the unique capability to generate much longer reads, thereby enhancing the overall sequencing potential. The ability of the MinION to produce longer amplicons significantly broadens the scope of biodiversity studies (Doorenspleet et al. 2021). Longer reads can capture more comprehensive genetic information, which can improve species identification, resolve complex taxonomic relationships, and provide deeper insights into the genetic diversity within ecosystems. Moreover, the extended read lengths can result in more reliable alignments and taxonomic assignments, reducing the likelihood of errors associated with shorter, fragmented sequences. The potential of the MinION is further enhanced by its real‐time sequencing capabilities, which allow for rapid data acquisition and analysis (Pomerantz et al. 2018; Srivathsan et al. 2024). This feature is particularly advantageous in field‐based studies, where timely results are crucial. By leveraging longer amplicons, MinION not only improves the quality and accuracy of the data but also enables a more efficient and effective approach to eDNA analysis, particularly for complex and diverse taxa such as insects (Doorenspleet et al. 2023; Harris et al. 2023). This technology holds great promise for advancing ecological research and biodiversity monitoring, offering a powerful tool for capturing and interpreting the genetic signals present in environmental samples. Furthermore, as the device is portable, it allows sequencing of the samples directly in the field and thus eventually adapts the sampling effort on site, based on the preliminary results obtained (Runtuwene et al. 2019). This approach may thus simplify and accelerate various management actions, such as protecting a site where an endangered or rare species has been detected or implementing eradication measures when a pest invasive species is found.

While our study provides a proof of concept of the application of the methodology, many aspects can be improved. The sampling method was not fully evaluated in detail—for example, by comparing it to manual sampling or other traditional methods. Moreover, the taxa accumulation curve based on our sampling indicates that we still need more samples to reach the entire insect diversity of the site. As done with eDNA sampling of water (Altermatt et al. 2023), sampling standardisation should be done to ensure low rates of false negatives (e.g., number of replicates taken, strategy for different types of vegetation, time and pattern of drone sampling). Also, while the Oxford nanopore technology starts to be useful for eDNA analyses and can speed up the entire process, the average accuracy remains restricted, and we could only retain a limited percentage of the reads with low error rates for taxonomic identification. Sequencing depth is also an important parameter for species detection in eDNA analyses (Bruce et al. 2021; Shirazi et al. 2021) and more exploration of sequencing depths should be performed to ensure optimal parameters. Our study paves the way toward a more systematic monitoring of global vegetation. In addition, while the insect primers were specific and well able to recover insects, many taxa were determined only to family or order. For an accurate taxonomic assignation, a complete reference database is needed. Thus, if no barcode sequence for a specific taxon was present in the reference database, we could not assign our sample(s) to that taxon. Furthermore, a region‐specific reference database would be usually necessary to obtain the best taxonomic results (Fueyo et al. 2024).

## 5 Conclusion

We believe that this approach has significant potential for broader applications, providing scalable and efficient means to evaluate the impact of sustainable agricultural practices on biodiversity. It demonstrates the potential of combining eDNA metabarcoding with innovative technologies such as drone‐based sampling, nanopore sequencing and plant‐surface DNA collection to monitor insect (or potentially other animals) biodiversity effectively. The diverse insect communities detected across various vegetation types not only highlight the ecological richness of grassland and shrubland environments but also underscore the capability of these methods to identify rare and significant species, including pests with potential impacts on agriculture and forestry. Furthermore, the integration of the MinION sequencing platform has proven valuable in enhancing the accuracy and efficiency of species identification through longer read lengths and real‐time sequencing. Despite the promising results, there remain several areas for improvement. The sampling effort needs to be optimised, and the standardisation of sampling protocols is essential to minimise false negatives. Additionally, the accuracy of the sequencing process could be further refined, and deeper exploration of sequencing depths is required to ensure optimal detection and species identification. Nonetheless, this study paves the way for future advancements in eDNA applications, offering a fast, scalable and non‐invasive approach to biodiversity monitoring that can be adapted to various ecosystems, including agricultural landscapes. As research in this field evolves, further optimisation and the development of comprehensive reference databases will enhance the potential of this technology in conservation and ecological studies. By expanding the use of our method, it could become a valuable tool for farmers, conservationists and policymakers aiming to enhance the ecological sustainability of agricultural landscapes.

## Author Contributions

**Darina Koubínová:** data curation (equal), formal analysis (equal), investigation (equal), methodology (equal), visualization (equal), writing – original draft (equal). **Steffen Kirchgeorg:** conceptualization (equal), data curation (equal), investigation (equal), methodology (equal), writing – original draft (equal). **Christian Geckeler:** data curation (equal), formal analysis (equal), methodology (equal), software (equal), writing – original draft (equal). **Sarah Thurnheer:** data curation (equal), formal analysis (equal), methodology (equal), validation (equal), writing – original draft (equal). **Martina Lüthi:** formal analysis (equal), methodology (equal), visualization (equal), writing – original draft (equal). **Théophile Sanchez:** data curation (equal), methodology (equal), software (equal), writing – original draft (equal). **Stefano Mintchev:** conceptualization (equal), funding acquisition (equal), investigation (equal), project administration (equal), supervision (equal), validation (equal), writing – original draft (equal). **Loïc Pellissier:** conceptualization (equal), funding acquisition (equal), investigation (equal), methodology (equal), project administration (equal), resources (equal), supervision (equal), validation (equal), visualization (equal), writing – original draft (equal). **Camille Albouy:** conceptualization (equal), data curation (equal), formal analysis (equal), investigation (equal), methodology (equal), project administration (equal), supervision (equal), validation (equal), visualization (equal), writing – original draft (equal).

## Conflicts of Interest

The authors declare no conflicts of interest.

## Data Availability

All the code and data will be available on the envidat platform https://doi.org/10.16904/envidat.643. For code and data review please see: https://github.com/Camillealbouy/su_ch_birmen_2023.
